# The History of a Case of Tuberculous Disease

**Published:** 1820-02

**Authors:** 

**Affiliations:** Physician to St. Mary's Hospital, and to the Royal Lunatic Asylum, at Konigsberg, in Prussia.


FOR THE LONDON MEDICAL AND PHYSICAL JOURNAL.

***The History of a Case of Tuberculous Disease.*** By Dr. WILLUDOVIUS, Physician to St. Mary's Hospital, and to the Royal Lunatic Asylum, at Konigsberg, in Prussia.

THE subject of this history was an unmarried woman, forty years of age, who had always enjoyed good health until the illness which immediately preceded her death. About six weeks before this took place, she complained, without presenting any well-determined signs of disease, of a constantly-increasing debility; still this was not sufficient to prevent her pursuing her usual active exercise, which her situation in life required. After some time a number of tubercles appeared just beneath the skin, throughout the body generally, but particularly about the neck, the breasts, and the abdomen, unattended by any pain, and without any alteration in the state of the skin itself. With this increasing debility, and the appearance of the tubercles above-mentioned, the patient at the same time experienced a sense of load and dull pain* in the region of the liver. The appetite was totally lost: the excretions were irregular and small in quantity. About this time an enlargement of the liver could be distinctly felt; and some of the tubercles under the skin had acquired the size of a small pigeon's egg. The patient soon became much emaciated; hectic fever appeared; the feet became œdematous; the abdomen became tense, and evidently contained fluid. A very severe cough and obstinate constipation of the bowels next afflicted the patient, and she at length fell a victim to her malady. Her death took place in the month of August, of this year, (1819,) which was certainly the hottest part of it; but, on the following day, when the dissection was made, the stench of the corpse was powerful, that I do not remember ever to have experienced any thing at all comparable to it.

The tumors under the skin were very hard, and were dispersed throughout nearly the whole of the surface of the body. They were not particularly situate about the inner surface of the limbs, where the lymphatic vessels run, neither were they the conglobate glands, for they were not found in the inguinal region and in the axillæ; and the salivary and parotid glands were unaltered in their structure. On the other hand, about the back, where, according to SOEMMERING, none of the glands above alluded to, exist, those tubercles were not wanting. The breasts themselves were not harder than usual, but, there were many tubercles beneath the skin of them. On making an incision into the tubercles above described, they presented a more or less hard, and even cartilaginous, substance, in the middle of which was a small quantity of thin fluid, which spurted out on the knife penetrating the surface retaining it.

The cavity of the abdomen contained a large quantity of extremely fetid, opake, and slimy fluid: the liver was unusually large, extending far into the left by hypochondre. On cutting into this viscus, I found in some parts a fatty, and in others a gelatinous, mass: the proper structure of its substance was but very small, the greater part of it being changed into the substance above designated. The gall-bladder contained a small quantity of greenish bile, and a very small gall-stone. The vena portarum were not varicose and in the ordinary state.

The stomach itself was, from the abolition of the nutritive function, small and shrivelled, especially about the cardiac and

---

* As this is so important a character in the history of the disease, we shall give the words of the original, for the satisfaction of those who are acquainted with the language: it is "Empfand die Kranke zugleich *Druck* und *dumpfen* Schmerz in der Lebergegend."—EDIT.

pyloric orifices. The spleen was large and soft; it contained, as well as the liver, a great number of separated sacs, containing a gelatinous mass.

The pancreas was not remarkably altered in structure. The mesenteric glands were very considerably enlarged, and, on cutting into them, they presented a tallow-like mass, containing in the middle a little thin fluid. Both with respect to bulk as well as to texture, the whole of those glands had undergone a similar alteration from the natural state. The kidneys were considerably softer in their texture than is usual, but they were free from the morbid organization above described. The ovaria were much enlarged: between them both, and near to the right one, was a large tumor of the size of a large man's fist, which contained a gelatinous and nearly transparent mass, intersected by a fine cellular tissue. The ovaries themselves were of a more tender, and rather drier texture, than ordinary, and contained some watery fluid.

The chest contained a small quantity of water; and, though the lungs presented some degree of alteration of structure, yet it was not so strongly marked as that of some of the organs already noticed. The bronchial glands were unaltered; and the heart was in the healthy state.*

* *Journal der Practischen Heilkunde*, xxxxviii. Band. Berlin.

